# Commissioning of a Monte Carlo‐based scanning proton beam for breast cancer: Incorporating LETd calculations and variable RBE models

**DOI:** 10.1002/acm2.70477

**Published:** 2026-01-27

**Authors:** Zhen Cao, Qing Zhang, Jingfang Zhao

**Affiliations:** ^1^ Department of Medical Physics, Shanghai Proton and Heavy Ion Center, Fudan University Cancer Hospital Shanghai Key Laboratory of Radiation Oncology, Shanghai Engineering Research Center of Proton and Heavy Ion Radiation Therapy Shanghai People's Republic of China; ^2^ Department of Radiotherapy The Third Affiliated Hospital of Naval Medical University Shanghai People's Republic of China; ^3^ Department of Radiation Oncology, Shanghai Proton and Heavy Ion Center, Fudan University Cancer Hospital Shanghai Key Laboratory of Radiation Oncology, Shanghai Engineering Research Center of Proton and Heavy Ion Radiation Therapy Shanghai People's Republic of China

**Keywords:** breast cancer, dose‐averaged LET, Monte Carlo, proton therapy, variable RBE model

## Abstract

**Background:**

Using a constant relative biological effectiveness (RBE = 1.1) in proton therapy may underestimate the RBE‐weighted dose in high linear energy transfer (LET) regions at the distal end of the beam, thereby limiting the ability to accurately predict clinical outcomes.

**Purpose:**

To commission and validate a Monte Carlo (MC) model incorporating variable RBE for breast cancer proton therapy, enabling improved RBE‐weighted dose calculation.

**Methods:**

A FLUKA‐based MC model of a raster scanning proton beamline was commissioned and benchmarked against the clinically employed treatment planning system (TPS) (Siemens Syngo) and physical measurements. Dose‐averaged LET (LET_d_) and variable RBE‐weighted dose distributions were computed using McMahon (McM), McNamara (McN), and Wedenberg (Wed) models. Treatment plans for four representative breast cancer cases were recalculated to compare TPS and MC results using dose‐volume histograms (DVH) and three‐dimensional gamma (γ) analysis. LET_d_‐volume histograms (LVH) and variable RBE‐weighted dose distributions were analyzed to compare cases without adverse effects versus those presenting rib fractures or radiation pneumonitis.

**Results:**

The FLUKA‐MC model showed good agreement with both the TPS results and the measured data, exhibiting proton range deviations within ±0.1 mm. The γ pass rates for the four patients are 94.0%, 92.2%, 92.6%, and 86.7%, respectively. LET_d_ analysis of 0.5 cm^3^ volumes of rib revealed numerical differences (fracture cases: 11.1 and 10.8 keV/µm; non‐fracture: 9.2 and 10.0 keV/µm). The RBE‐weighted dose to 0.5 cm^3^ of the ribs was consistently elevated in fracture cases across all models (RBE = 1.1: 46.2–49.0 Gy; McM: 54.6–56.5 Gy; McN: 51.0–53.3 Gy; Wed: 50.6–52.5 Gy) versus non‐fracture cases (RBE = 1.1: 44.0–45.3 Gy; McM: 52.2–53.8 Gy; McN: 48.6–50.1 Gy; Wed: 48.3–49.8 Gy). The estimated RBE values in the rib region were 1.60 (McM), 1.38 (McN), and 1.44 (Wed), which were derived from the mean LET_d_ within 0.5 cm^3^ rib volumes. The RBE‐weighted lung V20 was elevated in pneumonitis patients across all models. All variable RBE models predicted elevated RBE‐weighted doses in distal proton beam regions across cases.

**Conclusions:**

The commissioned MC framework demonstrated the feasibility of integrating multiple variable RBE models for RBE‐weighted dose estimation in proton therapy.

## 1 INTRODUCTION

Proton radiotherapy provides considerable advantages for breast cancer treatment, particularly for cases requiring post‐mastectomy or regional nodal irradiation, where the clinical target volumes (CTV) are close to organs at risk (OAR).^1^ The Bragg peak (BP) enables superior dose conformality, reducing radiation exposure to normal tissues.^1^, ^2^

However, most clinical practice relies on a constant relative biological effectiveness (RBE) of 1.1,^3^ despite evidence that RBE varies with linear energy transfer (LET), dose fractionation, and tissue type.^4^, ^5^, ^6^, ^7^ This variability raises concerns for breast cancer treatment, where high LET regions near the distal edge of the proton beam frequently overlap critical structures such as the ribs and lung parenchyma.^8^, ^9^ Applying a uniform RBE value may underestimate the RBE‐weighted dose to these OAR, potentially increasing toxicity risks including rib fractures^8^, ^10^, ^11^ and radiation pneumonitis.

Proton therapy centers increasingly integrate advanced strategies like LET/RBE‐optimized planning and retrospective dose analysis using variable RBE models into clinical workflows.^9^, ^12^, ^13^, ^14^, ^15^ The growing adoption of variable RBE models in proton therapy underscores the need for robust methods to compute RBE‐weighted dose distributions. A number of phenomenological RBE models have been published,^16^ derived from varying experimental datasets and underpinned by distinct biophysical assumptions. To date, however, there is no conclusive experimental or clinical evidence demonstrating the universal superiority of any single variable RBE model across tissues, dose levels, and LET conditions. Given this lack of consensus, model selection in this study prioritized formulations with established acceptance in clinical research methodological transparency. In this study we therefore focused on three representative variable RBE models: McMahon (McM),^17^ McNamara (McN),^18^ and Wedenberg (Wed)^19^ models. They were chosen based on their frequent citation and widespread adoption in recent treatment planning studies,^8^, ^15^, ^20^, ^21^, ^22^, ^23^, ^24^ which facilitates methodological comparability and interpretability of RBE‐weighted dose estimates. Their inclusion enables a robust comparison with the existing literature and enhances the clinical relevance of the variable RBE estimates presented in this work.

The McM model, which serves as a metric to account for the LET‐dependence of RBE rather than a fully parameterized biophysical RBE model, assumes a linear dependence relationship between LET and RBE and has been clinically applied in studies linking LET distributions with proton‐induced rib fractures in breast cancer.^8^ Separately, the general methodology of LET‐based optimization, which builds upon a similar linear LET‐RBE relationship assumption, has been applied to guide intensity‐modulated proton therapy (IMPT) reoptimization for intracranial tumors.^25^ Both the McN and Wed models are grounded in the linear‑quadratic (LQ) framework and share the key assumptions of a linear dependence of $\mathrm{RB}E_{\max}$ on LETd and an inverse relationship to the ${\left(\alpha /\beta \right)}_{x}$ ratio. These assumptions are particularly relevant for evaluating potential RBE enhancement in late‑responding tissues, including structures like the rib.^8^, ^26^, ^27^ While the two models differ slightly in their treatment of $\mathrm{RB}E_{\min}$, this discrepancy has been shown to exert limited influence on overall RBE estimates in clinical applications.^28^

When provided with identical physical dose and LET inputs, these models yield divergent RBE‐weighted dose estimates, as systematically demonstrated by Rørvik et al.^16^ This highlights the need for computational platforms that can simulate both the physical and biological components of proton interactions with high accuracy. Although commercial treatment planning systems (TPS) increasingly support LET calculation and variable RBE implementations,^29^, ^30^, ^31^ their closed‐source architecture and lack of standardized validation protocols restrict both detailed control of the underlying physics and reliable cross‐platform reproducibility. In contrast, the open‐source Monte Carlo (MC) code FLUKA provides the flexibility to simulate a broad spectrum of particles, energies, geometrical setups, and physical interactions.^32^, ^33^, ^34^ This flexibility makes FLUKA particularly valuable for independent validation of clinical treatment plans and for research on RBE modeling.

Independent research groups have demonstrated the capability of FLUKA to support advanced radiobiological modeling, including the integration of oxygen enhancement ratio (OER) effects^20^, ^35^ and LET‐guided recalculation workflows with variable RBE parameters.^21^ Building on these developments, our study aimed to establish and validate a FLUKA‐based MC framework that incorporates variable RBE model for breast cancer proton therapy. This framework was subsequently applied to four clinical breast cancer cases with rib fractures and pneumonitis, enabling an evaluation of variable RBE effects and an assessment of relationship between RBE‐weighted dose metrics and observed clinical toxicities.

## 2 MATERIALS AND METHODS

**FIGURE 1:**
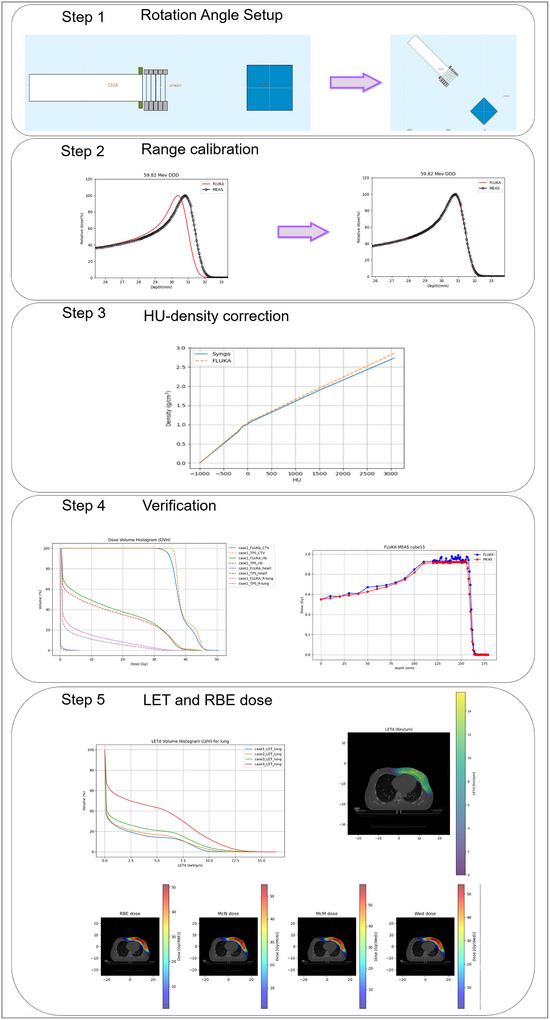
Step‐to‐step process overview of MC model: Rotating gantry angle setup, followed by range calibration. Next, the Hounsfield Unit (HU)‐density curve was calibrated, and dose verification was performed to ensure accuracy. Finally, the RBE was calculated with the calibrated model.

Commissioning and validation of the MC model were performed using the FLUKA MC code and the Siemens Syngo TPS. The Syngo TPS, equipped with a pencil beam scanning (PBS) algorithm and paired with a SIEMENS IONTRIS synchrotron to deliver proton in an energy interval ranging from 48.08 up to 221.07 MeV. As delineated in Figure 1, the workflow comprised sequential stages: initial beam model calibration (Steps 1–4) followed by LET_d_ and RBE dose calculations (Step 5). Through customized modification of FLUKA's user routines, three variable RBE models (McN, McM, Wed) were integrated into the simulation framework, enabling voxel‐level computation of biologically effective dose distributions.

These model outputs were systematically compared with the conventional constant RBE (1.1) approach using representative breast cancer treatment plans, with focused analysis on ribs and lungs as clinically critical organs‐at‐risk where LET_d_ and RBE variability are most clinically consequential.

### 2.1 Geometry and settings in FLUKA

The geometry and beam configuration were adapted from our previous research,^36^ with key modifications implemented to enhance clinical relevance. Specifically, we applied a 45‐degree gantry rotation and beam source to simulate beam delivery at clinically established angles. For improved accuracy, the ‘PRECISIO’ option was selected within the DEFAULTS card in FLUKA.

The particle emission direction and coordinates were adjusted according to Equations (1)–(4) implemented in the source.f file, enabling accurate modeling of particle emission from the 45‐degree gantry position.

(1) $$x_{pos}=x_{pre}$$

(2) $$y_{pos}=y_{pre}*\cos\left(\alpha \right)-z_{pre}*\sin\left(\alpha \right)$$

(3) $$z_{pos}=z_{pre}*\cos\left(\alpha \right)+y_{pre}*\sin\left(\alpha \right)$$

(4) $$y_{dir-pos}=y_{dir-pre}-\cos\left(\alpha \right)$$

where α is rotation angle, $x_{pre}$, $y_{pre}$, and $z_{pre}$ represent the original particle coordinates, while $x_{pos}$, $y_{pos}$, and $z_{pos}$ are the transformed coordinates. Similarly, $y_{dir-pre}$ is the original particle y‐direction cosine and $y_{dir-pos}$ is the modified cosine.

### 2.2 Range calibration

Building upon the method established in prior research,^36^ we employed a linear model to fit the simulated energy, a third‐order polynomial for the energy spread, and two fourth‐order polynomials to model the full‐width at half maximum (FWHM) in the X and Y directions.

A water tank with dimensions of 30 cm × 30 cm × 30 cm was modeled in FLUKA. Proton beams of 15 different energies, ranging from 48.08 to 221.07 MeV, were simulated to assess their range. The energies were adjusted using the energy‐range equation^37^:

(5) $$E\left(z\right)=\frac{{\left(R_{0}-z\right)}^{\frac{1}{p}}}{\alpha^{\frac{1}{p}}}$$

E(z) denotes the residual energy at depth z, for $z<R_{0},$ where E(0) is the initial energy corresponding to a range $R_{0}$ at z=0. By adjusting z, we were able to minimize the range error between FLUKA‐simulated and the measured value, achieving accuracy within 0.1 mm. Here, the range is defined as the depth at which 80% of the maximum dose is delivered distal to the BP. The constants a=0.0022cm/MeV, and p=1.77 were derived from the Bortfeld approximation.^37^

### 2.3 Calibration of HU‐Density

To establish a reliable Hounsfield Unit (HU)‐to‐density conversion for FLUKA simulations, we adopted a systematic approach by initially dividing the full HU range (−1000 to 3070) into 27 intervals, following established protocols.^38^, ^39^ Each interval was assigned a reference density corresponding to its median HU value using the Syngo TPS calibration curve,^21^ with voxel densities scaled relative to these reference values. However, to comply with FLUKA's density scaling constraints (requiring ratios between 2/3 and 3/2),^33^ we identified problematic HU ranges (−1000 to −950 and −950 to −120) where extreme density variations violated these limits. These intervals were subsequently subdivided into 12 smaller ranges, resulting in a final validated material map comprising 39 intervals.

In FLUKA, linear interpolation was employed to calculate the corresponding density for each HU value with each interval. The in‐house Python script was utilized to perform the linear interpolation and derive the corresponding FLUKA densities. Since discrepancies arose between the HU‐density values from Syngo TPS and those required for FLUKA simulations, we addressed this by dividing the Syngo density for each HU by the interpolated FLUKA density. This correction factor resulting from such a process was applied via the CORRFACT card in FLUKA. Finally, the validation of the calibration process was to utilize the correction factors to test tissue‐equivalent phantom containing various materials of known HU values and check that modified FLUKA calculations were consistent with expectations.

### 2.4 Verification in water phantom validation plans

Three proton treatment validation plans targeting depths of 5, 10, and 15 cm were simulated in FLUKA and experimentally measured using a water phantom (MP3‐P, PTW‐Freiburg). Dose profiles were recorded at 24 positions per depth plane with PinPoint ionization chambers (Model T31015, PTW‐Freiburg). The dose profiles obtained from the FLUKA simulations were compared with the measured data to verify accuracy.

### 2.5 Absolute dose calibration and patient recalculation

Absolute dose calibration was performed following the same method as in previous study.^36^ The proton beam model was validated using treatment plans from breast cancer patients receiving radiotherapy at a 45‐degree beam angle. Four patients who underwent breast‐conserving surgery were selected for the study. As of the most recent follow‐up, two patients with no adverse effects, one developed a rib fracture, and one suffered both a rib fracture and radiation pneumonitis.

Patients' DICOM Computed Tomography (CT) images and beam parameters were imported into FLUKA for MC simulations using 10^8^ particle histories to ensure statistical accuracy in dose calculations. The recalculated dose‐volume histograms (DVH) from FLUKA were then rigorously compared against the original Syngo TPS plans. Three‐dimensional gamma analysis (3%/3 mm) was performed using VeriSoft 7.0 (PTW‐Freiburg) with automatic alignment to correct for setup uncertainties. Low‐dose regions below 5% of the maximum dose were excluded from the analysis.

### 2.6 LET_d_ and RBE calculation

The LET_d_ and RBE was computed using FLUKA's comscw.f routine. In the dose scoring process, this routine applies a user‐defined scaling factor to each scoring bin when processing the deposited dose. For this study, the LET value for individual energy deposition events was obtained through calls to FLUKA's GETLET function. It was subsequently derived by averaging the pre‐ and post‐event LET values, implementing algorithm C as proposed by Cortés‐Giraldo et al.^40^ This algorithm determines LET_d_ based on comprehensive calculations of both energy and momentum before and after each energy deposition event. In line with recommendations for harmonizing LET reporting,^41^ our calculation included all proton secondary particles. The LET_d_ was defined in Equation (6). The *i* is every particle depositing energy in the volume, $d_{i}$ is the dose deposition of a particle and LETi is the LET from particle i.

(6) $$LET_{d}=\frac{\sum_{i}d_{i}LET_{i}}{\sum_{i}d_{i}}$$

The rib is classified as a late‐responding tissue with a low α/β ratio,^26^, ^27^ necessitating the selection of RBE models that account for both LET_d_ and α/β dependence to accurately capture RBE enhancement in low α/β tissues under high LET_d_ conditions. This rationale led to our inclusion of the McN model developed by McNamara et al.^18^ and the Wed model proposed by Wedenberg et al.,^19^ where methodological differences translate to clinically similar outcomes. Furthermore, the McM model by McMahon et al.^17^ was selected based on its established application in previous clinical studies investigating LET_d_ effects in proton‐induced rib fractures among breast cancer patients.^8^

The McM model was described by Equation (7):

(7) $$RBE=1+0.055*LET_{d}$$

Additionally, the McN model was utilized using Equations ((8), (9), (10)):

(8) $$\begin{array}{cc} & RBE\left[D_{p},\left(\alpha /\beta \right),LET_{d}\right]=\frac{1}{2D_{p}}\\ & \times \left(\sqrt{{\left(\alpha /\beta \right)}^{w}+4D_{p}\left(\alpha /\beta \right)RBE_{\max}+4D_{p}^{2}RBE_{{mix}^{2}}-\left(\alpha /\beta \right)}\right) \end{array}$$

(9) $$RBE_{\max}=0.99064+\frac{0.35605GY{\left(Kev/\mu m\right)}^{-1}}{\left(\alpha /\beta \right)}\times LET_{d}$$

(10) $$\begin{array}{cc} RBE_{\min} & =1.1012-0.0038703Gy^{-\frac{1}{2}}{\left(Kev/\mu m\right)}^{-1}\\ & \;\times \sqrt{\left(\alpha /\beta \right)}\times LET_{d} \end{array}$$

Here, Dp was fraction dose, and an α/β ratio of 3 Gy was applied, in line with the recommendations from previous studies.^8^

Finally, the Wed model was calculated using Equation (11):

(11) $$\begin{array}{cc} & RBE\left(D,LET_{d},\left(\frac{\alpha }{\beta }\right)\right)=-\frac{1}{2D}\left(\frac{\alpha }{\beta }\right)\\ & \;+\frac{1}{D}\sqrt{\frac{1}{4}{\left(\frac{\alpha }{\beta }\right)}^{2}+\left(qLET_{d}+\left(\frac{\alpha }{\beta }\right)\right)D+D^{2}} \end{array}$$

Where the *q* is 0.434 as recommended,^19^ and other parameters are consistent with the meaning of the McN model.

## 3 RESULTS

### 3.1 Range calibration in water

**FIGURE 2:**
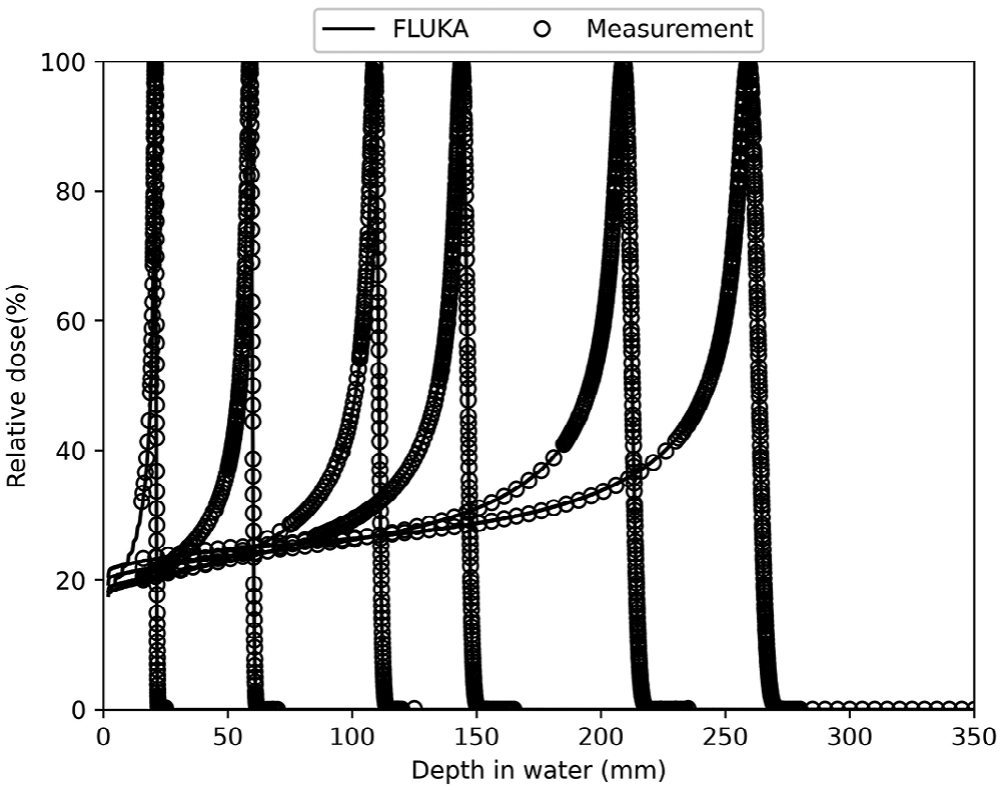
Simulated fit (line) and measured (dot) depth dose for proton beam energies of 48.08, 85.67, 121.08, 141.87, 175.72, and 199.03 Mev.

The simulations of proton beam range calibration were performed for 15 proton beam nominal energies in a water tank. The integral depth dose (IDD) curves derived from the FLUKA simulations were compared to physical measurements acquired at 45‐degree gantry angle using a PeakFinder device (PTW‐Freiburg, Germany) during system commissioning. Figure 2 presents relative depth‐dose curves comparing FLUKA simulations against measured data for six incrementally ordered proton beam energies—48.08, 88.67, 121.08, 141.67, 175.72, and 199.03 MeV, and a strong agreement was observed between the simulated and measured IDD curves. Across the 15 beam energies tested, the mean discrepancy between the FLUKA‐simulated beam ranges and measured values was within 0.1 mm. In terms of beam spot size, the comparison between the TPS and MC simulations revealed a maximum difference of 4.74%, with an average deviation of 2.9%, and most of the differences remained under 4%.

**FIGURE 3:**
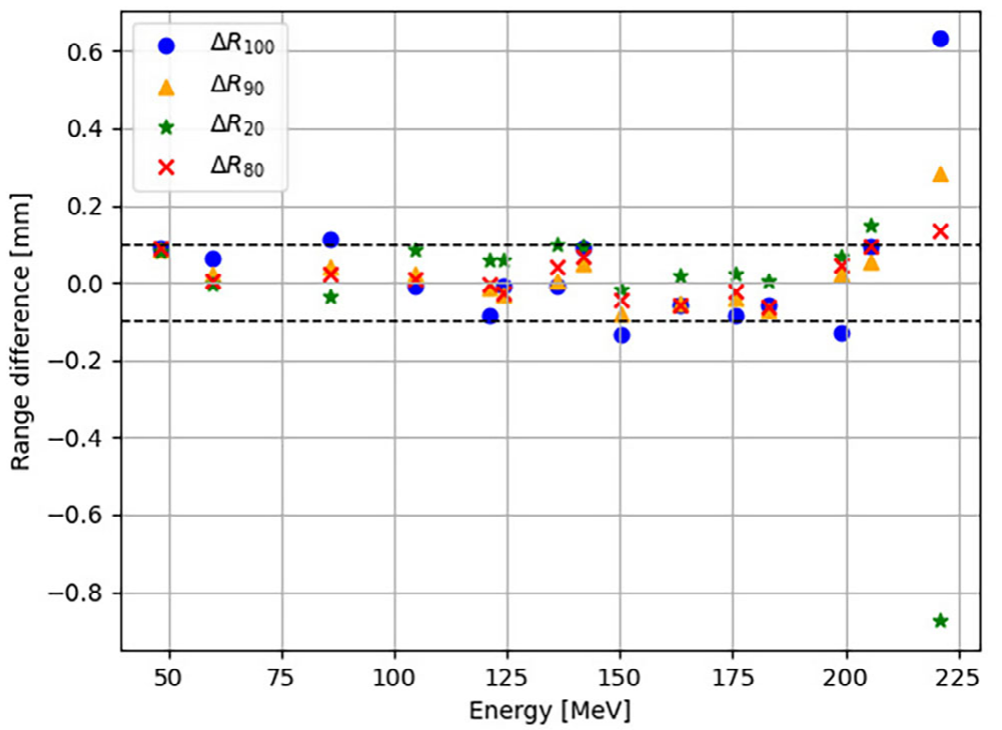
Range differences for the MC minus the TPS BP in mm for increasing energy, with markers indicating R100 (BP position), R90, R80, and R20 (distal to BP at 90%, 80%, and 20% dose levels, respectively). Black lines denote the ±0.1 mm threshold.

The range differences for the 15 energies, including the BP position (R100), 90% distal to the BP (R90), R80, and R20, are displayed in Figure 3. The black dashed line represents the standard tolerance of 0.1 mm. The energy ranges displayed minimal differences, falling within the standard tolerance of 0.1 mm, with an average range shift of just 0.04 mm. The biggest range (R80) variation obtained at 205.68 MeV is 0.097 mm.

### 3.2 Validation and commissioning for different tissue‐equivalent phantoms

**FIGURE 4:**
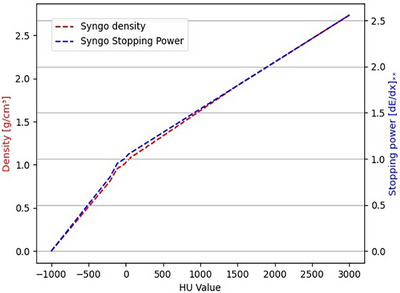
Density and stopping power ratio corresponding to HU values in Syngo TPS.

**FIGURE 5:**
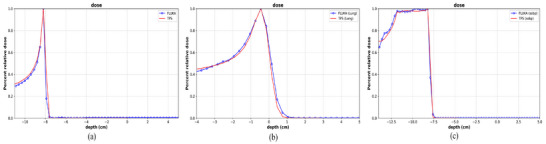
Depth dose curves comparing FLUKA simulations and measurements at the same location. (a) monoenergetic proton beam 85.67 Mev in water, (b) monoenergetic proton beam 98.02 Mev in lung‐equivalent phantom, (c) SOBP in water‐equivalent phantom.

Since HU was directly related to tissue density, which influences the range of the proton beam when imported into the CT images, an HU‐density correction was applied. The stopping power coefficients corresponding to various HU values were obtained from the Syngo TPS, and the corresponding densities were calculated using the method described by Schneider et al.^42^ (Figure 4). Following the correction of the density values based on this profile, validation plans for different tissue‐equivalent phantoms were imported, and FLUKA simulations were performed. These simulations were compared with one‐dimensional dose profiles. The results, including monoenergetic proton beams for water and lung‐equivalent validation plans and a spread‐out BP (SOBP) for the water‐equivalent phantom, are presented in Figure 5. The depth‐dose curves from the FLUKA simulations closely matched the TPS results.

**FIGURE 6:**
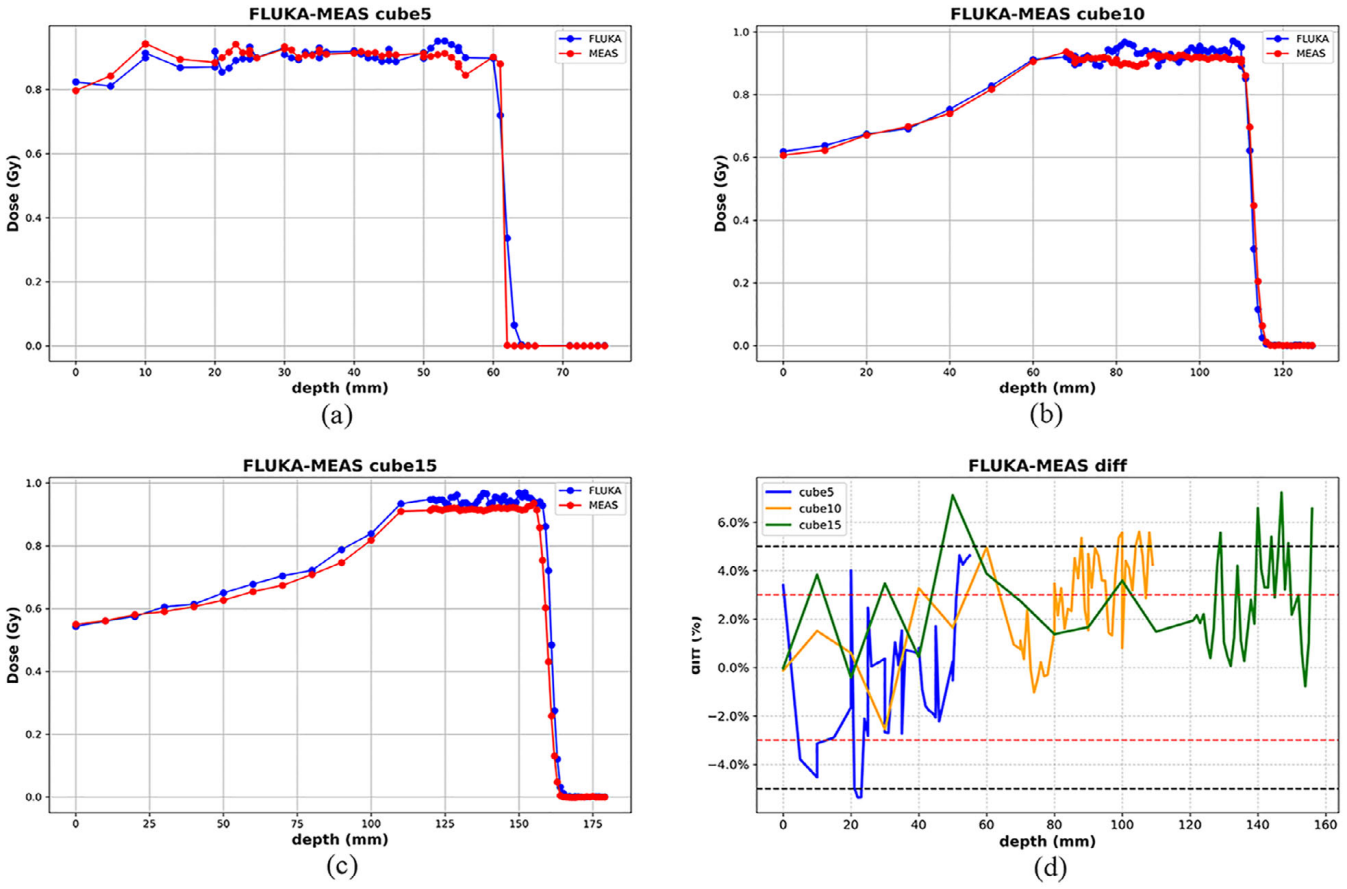
Comparison of one‐dimensional dose curves between FLUKA simulated doses and measured doses in three water verification plans at different depths in the target area (5 cm * 10 cm); (a) Depth of 5 cm, (b) Depth of 10 cm, (c) Depth of 15 cm, (d) Difference in FLUKA dose and measured dose.

The validation process was further extended to three different water phantoms at varying depths. The one‐dimensional depth dose curves of FLUKA simulated dose and measured dose are displayed in Figure 6.

### 3.3 RBE‐weighted dose and LET_d_ recalculation results for patient treatment plans

**FIGURE 7:**
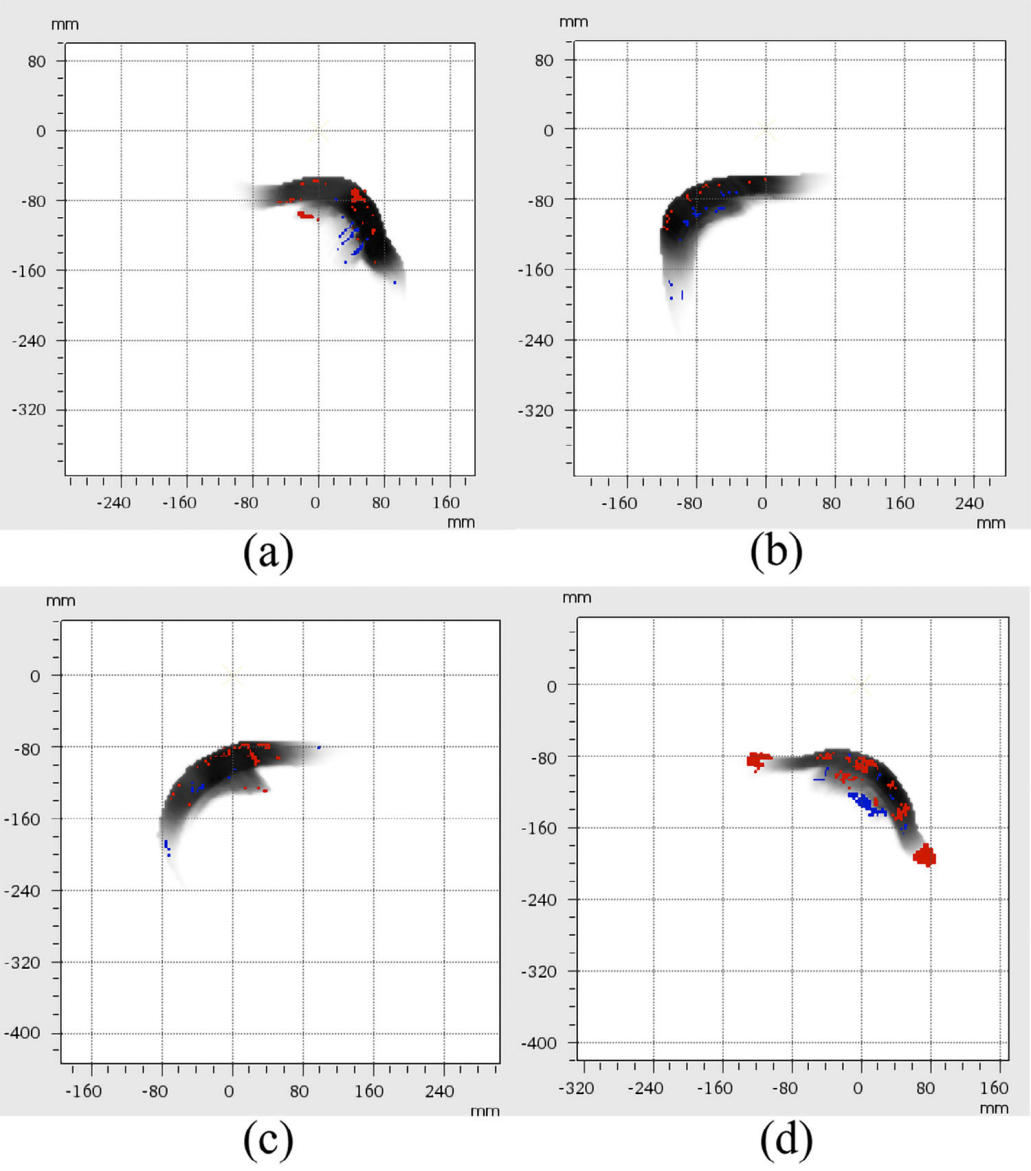
Two‐dimensional Gamma analysis of four patient cases, with red spots indicating hot points and blue spots indicating cold points (TPS minus MC calculation); (a) Case 1; (b) Case 2; (c) Case 3; (d) Case 4.

Four typical cases with at least 5 years follow‐up were selected, two cases presented without apparent radiation‐induced side effects, one presented with rib fracture, and one presented with both rib fracture and radiation induced pneumonia. Three‐dimensional γ analysis (3%‐3 mm) was performed for four patient cases using PTW VeriSoft 7.0 (PTW‐Freiburg) to compare the RBE dose (RBE = 1.1) distributions between TPS calculations and FLUKA simulations. To ensure robust analysis, dose volumes receiving less than 5% of the maximum dose were excluded, and auto‐alignment was applied to mitigate minor setup uncertainties during measurements. The γ‐analysis of four patient cases revealed pass rates were 94.0%, 92.2%, 92.6%, and 86.7% for the clinical treatment plans, respectively. Figure 7 presents the two‐dimensional γ analysis slices for four cases. Notable deviations occur predominantly at interfaces between tissues of markedly different densities, such as the lung–bone and lung–muscle boundaries, and in areas exhibiting large range variations along the oblique beam path. Specifically, Figure 7(a, b) show remarkable deviations at the lung‐bone/rib interface, Figure 7(c) demonstrates major discrepancies in areas of substantial soft‐tissue density variation, while Figure 7(d) exhibits distinct deviations at both the lung‐bone/rib interface and regions with large soft‐tissue density variations.

**FIGURE 8:**
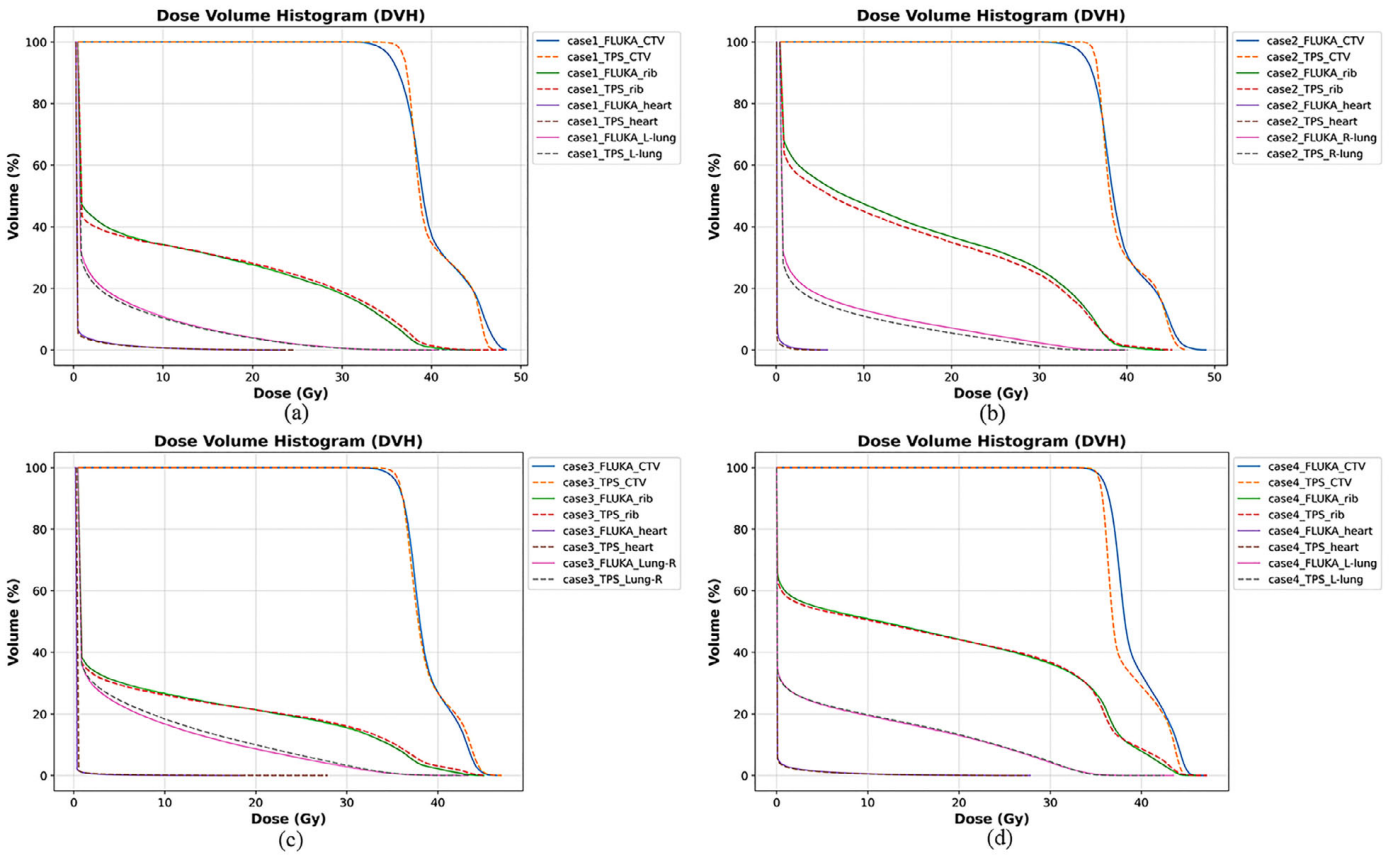
DVH curves for target volume and OAR in the clinical cases; (a) case 1, (b) case 2, (c) case 3, (d) case 4.

**FIGURE 9:**
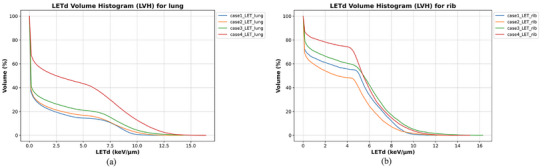
LVH curves for lungs ipsilateral to the target volume and ribs in the clinical cases; (a) LVH for lungs, (b) LVH for ribs.

Figure 8 shows DVH for the CTV and OAR, while Figure 9 presents LVH for ipsilateral lung tissue and ribs. The analysis showed moderately higher rib LET_d_ values in fracture cases (Case 3: 11.1 keV/µm; Case 4: 10.8 keV/µm) compared to non‐fracture cases (Case 1: 9.2 keV/µm; Case 2: 10.0 keV/µm) for the 0.5 cm^3^ rib volume. The LVH analysis further indicated a trend where patients who developed pneumonitis (case 4) had their lung LVH curves shifted toward higher LET_d_ values compared to those without pneumonitis (case 1–3). These elevated RBE‐weighted doses correspond to estimated rib RBE values of 1.60 (McM), 1.38 (McN), and 1.44 (Wed), derived from the mean LET_d_ within the 0.5 cm^3^ rib volumes.

**FIGURE 10:**
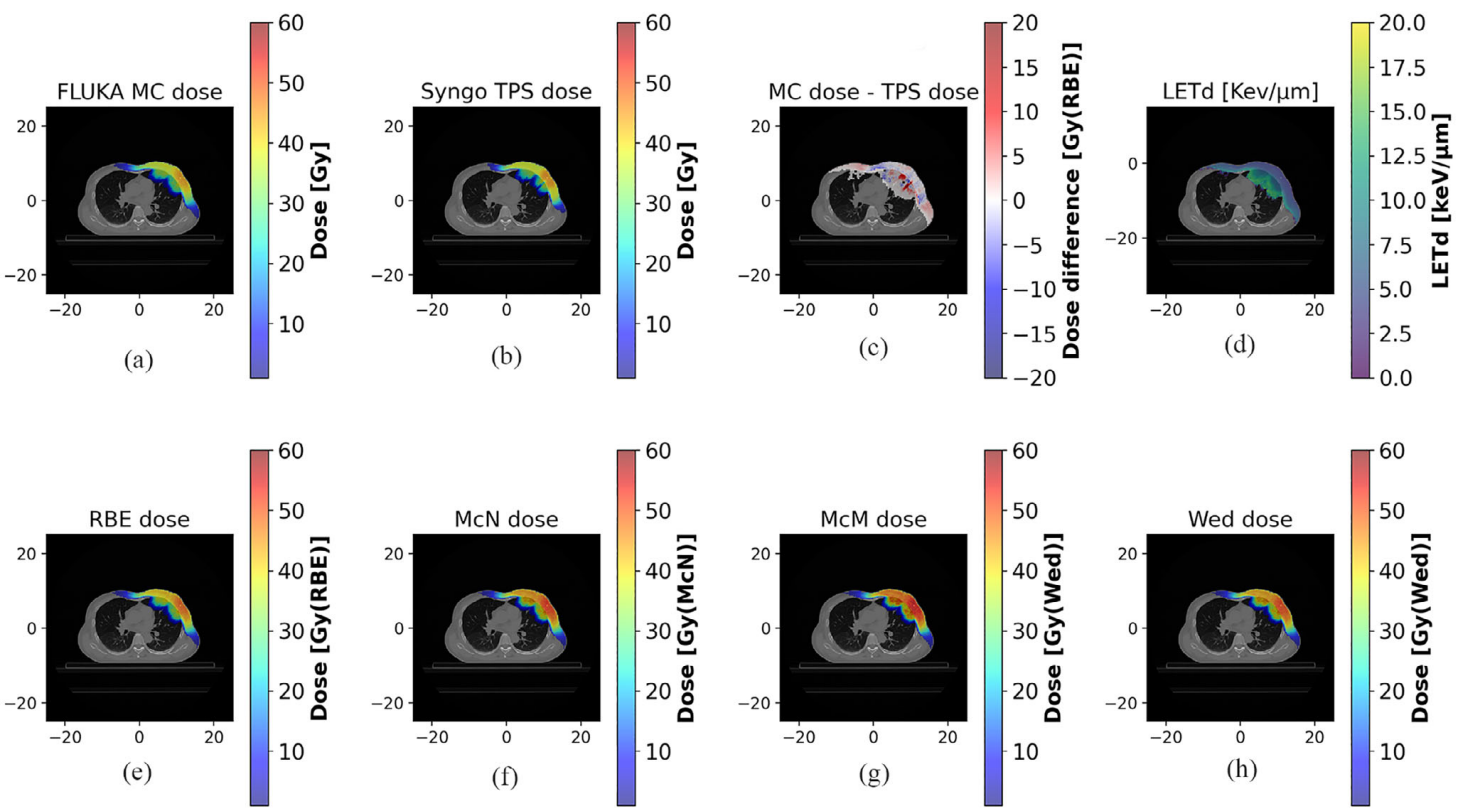
Comparison of physical dose and three RBE‐weighted dose distributions for the clinical case 4. All dose distributions are displayed for the total accumulated treatment course. (a) FLUKA Physical Dose, (b) TPS Physical Dose, (c) RBE‐weighted dose difference (FLUKA vs. TPS), (d) Dose‐Averaged LET (LET_d_), (e) RBE‐Weighted Dose (RBE = 1.1), (f) RBE‐Weighted Dose (McN Model), (g) RBE‐Weighted Dose (McM Model), (h) RBE‐Weighted Dose (Wed Model).

Figure 10 provides a detailed analysis for Case 4, comparing FLUKA‐simulated and TPS‐calculated physical dose distributions (a‐b), their differences (range: −10.9 to 18.4 Gy (RBE)) (c), LET_d_ distribution (d), and RBE‐weighted doses under four models (e–h). The physical dose difference map (c) quantifies localized discrepancies at tissue interfaces, particularly at the lung‐bone/rib interface, where 13 pixels exhibited differences exceeding 10 Gy and an additional 36 pixels had differences between 5–10 Gy, contrasting with sub‐5 Gy differences in homogeneous regions. Consistent with fundamental proton physics, the LET_d_ distribution (d) shows characteristic elevation at the distal end of the proton beam. The LET_d_ within the CTV remained below 7 keV/µm, while values in the distal lung and rib reached **∼**10 keV/µm.

**FIGURE 11:**
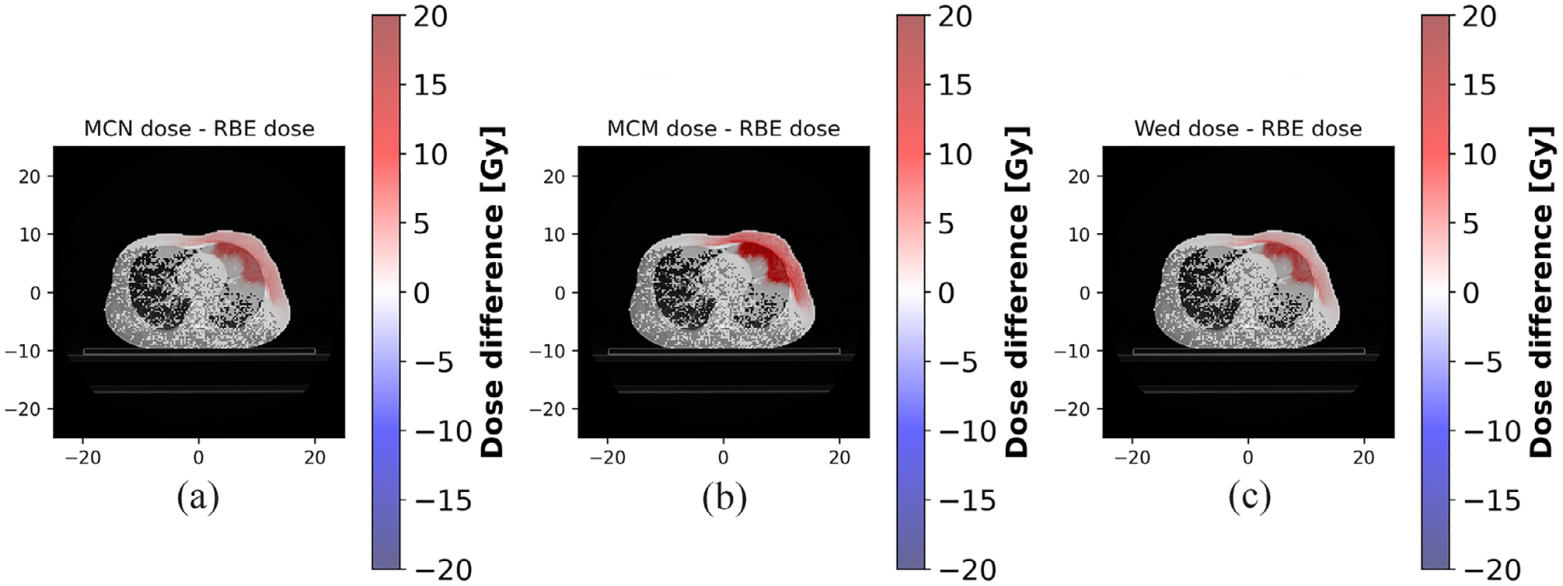
Inter‐model comparisons of variable RBE‐weighted dose for Case 4; Differences are shown for the total accumulated RBE‐weighted dose; MCN dose, MCM dose and Wed dose are the three variable RBE‐ weighted dose distributions described above; RBE dose is the dose distribution at an RBE of 1.1; (a) ΔDose (McN—RBE), (b) ΔDose (McM—RBE), (c) ΔDose (Wed—RBE).

This LET_d_ elevation directly resulted in increased RBE‐weighted dose predictions. All variable RBE models (f–h) calculated elevated RBE‐weighted doses in these high‐LET regions compared to the constant RBE = 1.1 model (e), with the most substantial increase predicted by the McM model. This model‐specific enhancement is quantified in Figure 11, which shows the differential RBE‐weighted dose for each variable model against the constant RBE baseline, with differences ranging from −0.0 to 7.3 Gy for the McN model, from −0.2 to 14.1 Gy for the McM model, and from −0.9 to 8.5 Gy for the Wed model. The magnitude of this effect is substantial; for the 0.5 cm^3^ rib volume in Case 4, the RBE‐weighted dose increased from 49.0 Gy (RBE = 1.1) to 56.5 Gy (McM), 53.3 Gy (McN), and 52.5 Gy (Wed). Similarly, for the lung, the RBE‐weighted V20 in the pneumonitis patient was elevated across all models (RBE = 1.1: 14.3%; McM: 17.5%; McN: 16.2%; Wed: 16.4%) compared to non‐pneumonitis controls (RBE = 1.1: 6.3%, 8.7%, 10.4%; McM: 8.9%, 11.3%, 13.9%; McN: 7.7%, 10.2%, 12.5%; Wed: 7.9%, 10.3%, 12.7%).

In summary, this pattern of distal RBE enhancement was consistently observed across all cases, with the magnitude of increase varying by the choice of model and the specific anatomy. Although the variable RBE models diverged significantly in high‐LET regions, they consistently converged back to RBE = 1.1 in the low‐LET beam entrance region. This phenomenon is supported by its consistent appearance in the comprehensive analysis of all other cases (Appendix Figures 1–3 for LET_d_/RBE distributions; Appendix Figures 4–6 for RBE dose differences).

## 4 DISCUSSION

This study established and validated a FLUKA‐based MC framework for breast cancer proton therapy, integrating three variable RBE models (McM, McN, and Wed). The framework achieved high physical accuracy, with proton range deviations within ±0.1 mm in a homogeneous water phantom, consistent with the AAPM clinical standards,^43^ and HU–density corrections ensuring tissue heterogeneity accuracy. Water phantom and absolute dose calibration confirmed the dosimetric reliability. Notably, this work represents the implementation of a FLUKA‐MC framework specifically for breast cancer, capable of computing LET and RBE‐weighted dose distributions across multiple models. Our model provides a flexible and fully parameterized platform for future cross‐model comparisons and validation studies.

Benchmarking studies show that phenomenological RBE models yield substantial, clinically relevant differences in predicted RBE‐weighted doses, particularly near the distal BP.^16^, ^44^, ^45^ Our patient analyses mirror these variations: rib‐fracture cases demonstrated moderately higher LET_d_ within 0.5 cm^3^ rib volumes and correspondingly higher RBE‐weighted doses across all three models, while pneumonitis patients showed elevated LET_d_ and increased RBE‐weighted V20 in lung LVH regions. Elevated RBE‐weighted doses at the rib–lung interface further reflect end‐of‐range biological effects and identify areas where tissue radiosensitivity may be underestimated by fixed‐RBE planning. These observations are consistent with prior variable‐RBE studies,^41^, ^46^, ^47^, ^48^ which reported higher RBE‐weighted doses than the clinical RBE = 1.1 assumption in distal high‐LET regions. Together, the benchmarking results, patient‐specific dose correlations, and localized LET‐dependent patterns indicate that variable RBE modeling reveals clinically important heterogeneities obscured by fixed‐RBE methods.

The clinical relevance of these findings is reinforced by our rib and lung analyses, which consistently associate elevated LET_d_ and RBE‐weighted dose with observed toxicities. Although LET‐guided optimization studies suggest potential reductions in normal‐tissue complications,^41^, ^46^, ^49^ optimization was not applied here. Instead, our results provide quantitative, patient‐specific benchmarks for understanding how different RBE models shape dose distributions and toxicity risk, laying the groundwork for future LET/RBE‐guided planning and clinical validation.

The integration of variable RBE models into MC dose engines has been implemented previously in TOPAS, GEANT4, and FLUKA platforms,^50^, ^51^, ^52^ and numerous studies have compared single model and multi‐model predictions in various scenarios.^16^, ^28^, ^46^, ^53^ In this context, our study extends prior work by focusing on breast cancer and providing multi‐model RBE calculations within a single, open, reproducible FLUKA framework. While TPS implementations increasingly support LET_d_ calculation and variable RBE‐based optimization,^29^, ^30^, ^31^ the closed‐source nature of commercial systems limits transparency, reproducibility, and detailed inter‐model comparison. By contrast, FLUKA allows full parameterization of particle types, energies, geometries, and RBE models, making it an ideal platform for independent validation and benchmarking.

In our study, Case 4 showed a lower γ‐passing rate than other cases, mainly due to limitations of the pencil beam (PB) algorithm in heterogeneous tissues. As reported by Jatinder et al.^54^ and Lomax AJ et al.,^55^ the PB algorithm assumes material homogeneity along beam paths, leading to dose inaccuracies at tissue interfaces (e.g., lung‐bone) and underestimation of distal fall‐off widths for oblique beams. Clinically, this results in large dose uncertainties, consistent with our findings: Figure 7(d) shows hot/cold spots at density transition zones within the target and at lung‐bone interfaces, reducing γ‐compliance. In contrast, Cases 1–3 (Figures 7(a–c)) with more uniform tissue density maintained higher γ‐passing rates. Liang et al.^56^ also found that PB algorithms overestimate target doses in breast IMPT plans, as breast tissue heterogeneity strongly affects proton range and dose distribution.^57^ Figure 7 confirms this, showing slightly elevated target doses calculated by the TPS.

The study also extends its findings by proposing a framework for integrating variable RBE models into 45‐degree gantry configurations. Rotating gantries, increasingly used in proton therapy centers, offer greater flexibility in beam angle selection, allowing for improved dose conformality and individualized treatment plans.^58^ However, the integration of a rotating gantry does add complexity to physical dose modeling. Variable beam direction, in conjunction with variable RBE, complicates commissioning and validation of treatment plans, prompting us to propose a new model to address these challenges. This establishes a methodological pathway for evaluating LET and RBE distributions in complex treatment geometries, thereby complementing ongoing research in LET‐driven and variable RBE optimization.^46^, ^47^, ^48^, ^59^, ^60^, ^61^ Despite computational intensity limiting direct clinical application, the framework serves as a high‐fidelity benchmarking tool for TPS validation, supporting more reliable clinical proton therapy planning.

Uncertainties in α/β ratio substantially impact RBE predictions in McN and Wed models, particularly in high‐LET regions.^16^, ^62^, ^63^ These uncertainties remain an intrinsic challenge in variable RBE modeling, and our study is similarly limited by the current lack of tissue‐specific α/β measurements for the breast cancer cohort. Incorporating these uncertainties into future analyses may help refine clinical risk estimation and improve model interpretability. Consistent with clinical studies, we adopted α/β = 3.0 Gy for the rib.^8^ The observed discrepancies between variable‐ and fixed‐RBE doses underscore the risk of underestimating normal tissue toxicity when assuming a constant RBE, supporting personalized RBE‐guided planning.^64^

Limitations of this study include the small breast cancer cohort and the need for broader testing across tumor sites. Computational demand remains a challenge, highlighting the need for algorithmic acceleration and workflow integration. Future work will expand clinical validation and improve computational efficiency.

## 5 CONCLUSION

This work establishes a validated MC framework incorporating variable RBE calculations for proton therapy, enabling 45‐degree treatment plan recalculation. This RBE modeling approach provides flexibility for evaluating RBE‐weighted dose distributions and supports retrospective analysis of clinical outcomes. However, to fully translate this methodology into clinical practice, further optimization is required to improve computational efficiency, and extensive clinical validation remains necessary to correlate model predictions with patient outcomes.

## AUTHOR CONTRIBUTIONS

Zhen Cao performed the experimental procedures and contributed to manuscript writing and revision. Jingfang Zhao designed the experimental details and contributed to manuscript writing and revision. Qing Zhang reviewed and revised the manuscript.

## CONFLICT OF INTEREST STATEMENT

The authors have no relevant conflicts of interest to disclose.
